# Supplementary material for: Ultra-high resolution imaging of thin films and single strands of polythiophene using atomic force microscopy
Source: Nat Commun. 2019 Apr 4;10:1537. doi: 10.1038/s41467-019-09571-6 (PMC6449331; doi:10.1038/s41467-019-09571-6)
Supplement: Supplementary file 1 — Supplementary Information [file 41467_2019_9571_MOESM1_ESM.pdf]

Supplementary Information

Ultra-high resolution imaging of thin films and single strands of polythiophene using atomic force microscopy

Korolkov et al.

Supplementary Methods: Computational details

All on surface structures were optimized with B3LYP exchange-correlation functional and 4-31G-1d basis set. To include dispersion correction, we used the Grimme method to optimize on-surface structures of both bithiophene (Supplementary Figure 1) and terthiophene (Figure 2). The basis set was chosen as it correctly reproduces both the crystal lattice of hBN (2.506 Å (calculated) vs 2.504 Å (experimental)) and molecular geometries when compared to available crystal structures. The geometry optimization was performed with fixed coordinates of B, N and H atoms of BN slab.

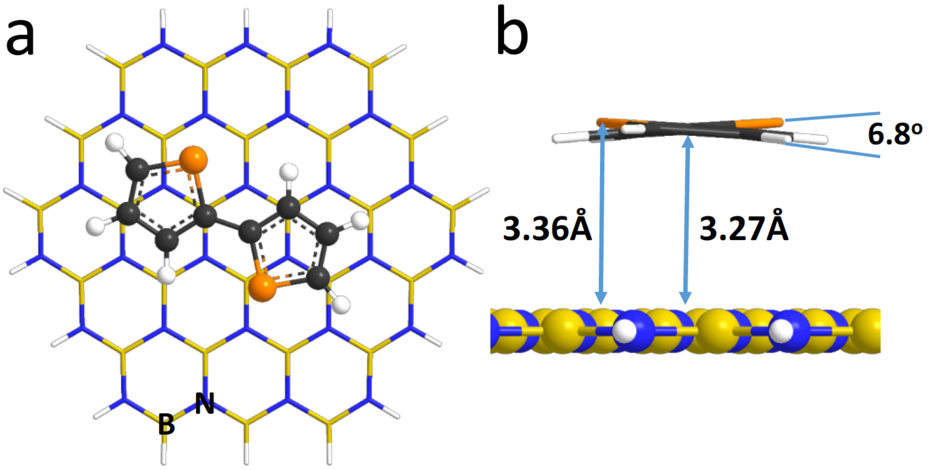

Supplementary Figure 1. Trans-gauche conformation of bithiophene adsorbed on a hBN slab. (a) – top view, (b) – cropped side view along the molecular axis showing rotational distortion of the molecule. Both the calculated torsional angle between thiophene units, and the molecule–substrate distances are shown.
